# Patient engagement in perioperative settings: A mixed method systematic review

**DOI:** 10.1111/jocn.16709

**Published:** 2023-04-18

**Authors:** Zahra Cooper, Sonja Cleary, Wanda Stelmach, Zhen Zheng

**Affiliations:** ^1^ School of Health and Biomedical Sciences, College of Science, Technology, Engineering and Mathematics (STEM) RMIT University Victoria Bundoora Australia; ^2^ Department of Surgery Northern Health Victoria Epping Australia

**Keywords:** patient actions, patient–clinician communication, perioperative patient engagement, shared decision‐making, surgical patient engagement

## Abstract

**Background:**

Patient engagement has emerged as a key focus in the research literature to facilitate patients' recovery. The term is commonly used by researchers, yet without working definitions. This lack of clarity is further complicated by the interchangeable use of a few terms.

**Objectives:**

This systematic review aimed at identifying how patient engagement was conceptualised and operationalised in perioperative settings.

**Methods:**

MEDLINE, EMBASE, CINAHL and the Cochrane Library were searched for publications in English discussing patient engagement during the perioperative phase. Three reviewers conducted study selection and methodological assessment using Joanna Briggs Institute mixed methods review framework. Reflexive thematic analysis was used to analyse qualitative data and descriptive analysis for quantitative data.

**Findings:**

Twenty‐nine studies were included with a total sample of 6289. Study types included qualitative (*n* = 14) and quantitative (*n* = 15) with different types of surgery. Sample sizes ranged from *n* = 7 to *n* = 1315. Only 38% (*n* = 11) of included studies offered an explicit definition. Four themes associated with operationalisation included provision of information, which was most studied theme, communication, decision‐making and action‐taking behaviours. All four themes were interconnected and co‐dependent on each other.

**Conclusions:**

Patient engagement in perioperative settings is a complex and multifaceted concept. The conceptual void in the literature calls for more theoretically informed and comprehensive approaches to researching surgical patient engagement. Future research should aim to better understand the factors that influence patient engagement, as well as the impact of different forms of engagement on patient outcomes through the whole surgical journey of a patient.

**What this paper adds to this topic**

- This review identifies an absence of a definition of patient engagement in perioperative settings.
- Four key themes are associated with operationalisation of patient engagement, including the provision of information, communication, decision‐making and action‐taking.
- There are strong intersections or interplays of the four elements, yet most research only focussed on one or two themes or on part of the surgical journey, without studying the whole patient journey.

## 1 INTRODUCTION

Internationally, patient engagement in direct health care, has been described as the ‘holy grail’ or equivalent to ‘a blockbuster drug of the century’ (Chase, 2012). A substantial body of literature pertaining to patients with chronic diseases shows that engaging patients in their health care leads to improved quality of life and physical health (Druss et al., 2010), and reduced hospital and emergence readmission (Greene & Hibbard, 2012). Surgery is a critical aspect of health care and is recognised by patients as being one of the most powerless events while hospitalised (Bickmore et al., 2009). General anaesthesia in surgery is associated with increased hospital length of stay (Neuman et al., 2014), lengthier stay in postanaesthesia care (Gabriel et al., 2017; Ganter et al., 2014) and postoperative complications such as postoperative pain (Ganter et al., 2014) and postoperative nausea and vomiting (PONV) (Apfel et al., 1999). There are significant gains for patients and for the healthcare organisation in examining whether and how patients are engaged in their recovery and in their perioperative journey.

Patient engagement is a term commonly used by researchers, yet with limited discussion on its working definitions. In the literature, patient engagement covers a wide range of activities, including engaging patients in policymaking, service design, governance, research and patient own care. This may have contributed to the varied definition. This situation is complicated by the use of interchangeable or similar terms, for example patient involvement, patient participation, highlighting the ambiguity and contested nature of the term (Gallivan et al., 2012; Graffigna & Barello, 2022; Manafo et al., 2018).

Furthermore, how patients engage with their health care could differ depending on the types of healthcare setting. A systematic review by Davis et al. (2007) reported that type of healthcare setting was one of five core factors that influenced the notion of patient engagement. Inpatients in emergency care settings had less opportunities for engagement due to uncertainty and lack of knowledge about their condition, compared with patients in ambulatory care settings. The relationship between patient and healthcare provider in primary care also differs from those in secondary or tertiary settings. Primary care providers often follow a patient's development and medical history for several years; and at the same time, patients may be able to exercise more autonomy as opposed to that in tertiary care settings. Patients found communication more difficult in a hospital setting when compared with their doctor's clinic (Davis et al., 2007). A systematic review revealed that there are significant gaps in knowledge regarding patient engagement in the hospital setting (Prey et al., 2014), in particularly in perioperative settings.

One issue that has been addressed in the research on patient engagement is the lack of consistency and interchangeable use of terms related to this concept (e.g. patient engagement, participation, empowerment and activation). While this review is not necessarily calling for a static, unidimensional definition of patient engagement, it calls for a need for greater awareness of the nuances of terminology and the interchangeable nature of terms. Some of these concerns have been voiced in other studies (Harrington et al., 2020; Manafo et al., 2018).

We could identify only two definitions about patient engagement that have been applied to surgical settings (Law et al., 2022). Graffigna et al. (2016) proposed Patient Health Engagement from a psychosocial perspective, which illustrates a four‐stage dynamic engagement journey from feeling ‘blackout’ or ‘disengaged and overwhelmed’ about the diagnosis, to the arousal stage of experiencing illness, then identifying as a patient of ‘adhesion’ stage and reaching the ‘eudaimonic’ stage in which patient adjusts to the health condition and manage it. This definition was welcomed by surgical registers and helped junior doctors understand at which stage patients are in their engagement journey (Graffigna & Barello, 2018). The authors developed a measurement tool that has been included in ‘Clinical guidelines on perioperative management strategies for enhanced recovery after lung surgery’ (Gao et al., 2019, p. 1174). Hibbard et al. (2004) focussed on patient activation aspect of the engagement through understanding patients' self‐perceived efficacy and confidence. The associated Patient Activation Measure has been used in 10 papers about surgical patients and found that patients with higher level engagement are more likely to report better physical health after surgery and greater satisfaction (Law et al., 2022).

Neither of the two definitions clearly identify the full picture of patient engagement during perioperative stage. There is no research that has attempted to synthesise different ways patient engagement being conceptualised and operationalised in perioperative care settings, representing a considerable gap in the literature.

The research question was ‘*How is patient engagement conceptualised and operationalised in peri‐operative period*?’ and addressed through systematically reviewing qualitative, quantitative or mixed methods research. Conceptualisation refers to how the researchers defined patient engagement in their research, whereas operationalisation refers to specific elements or interventions that were studied. The objectives of the review were to explore:

1. how surgical patient engagement during the perioperative period was conceptualised, and
2. what patient engagement themes appeared in the operationalisation of this concept.

## 2 METHODS

This is a mixed method systematic review. The decision was made to include insights both from research that collected qualitative and/or quantitative data in order to give a more holistic representation of the broad themes that encompassed research discourses in this space. This review was registered with The International Prospective Register of Systematic Reviews (*PROSPERO CRD42020178688*).

### 2.1 Search strategy

Searches across MEDLINE, EMBASE, CINAHL and the Cochrane Library were conducted, using a combination of free text and MESH terms on Medline. A two‐part terminology search was used to identify studies that (1) discussed patient engagement (2) focussed on surgical patients in perioperative settings. Search terms and strategies are provided in Appendix S1. To be included, studies must (1) report patient engagement data from patient perspective; (2) report data collected during perioperative phases, that is 2 weeks prior to the surgery to 2 weeks after surgery; and (3) include adult patients who were able to provide informed consent undergoing surgical procedures under general anaesthesia. Studies were limited to those in English and in peer‐reviewed journals. Studies were excluded if (1) patient engagement was about in policymaking or research; (2) surgeries were associated with special needs and considerations that differ from usual operations, including cancer, dental, organ transplant, obstetrics and IVF; (3) data were collected only from rehabilitation setting, home care or out of hospital setting; and (4) no patient data were provided.

Abstracts were screened ZC and reviewed by ZZ (quantitative studies) and SC (qualitative studies). Full texts for eligible studies were retrieved and further assessed by all three reviewers. The following data were extracted: (a) study design and methodology, (b) setting/context, (c) target population, (d) type of surgery, (e) working definition of patient engagement, (f) interventions (if applicable) and (g) study limitations.

### 2.2 Quality appraisal

Each study underwent a quality appraisal, using relevant checklists in JBI Critical Appraisal Tools. Five JBI tools used to assess the quality of studies based on research design: randomised control trials, quasi‐experimental studies, cross‐sectional analytical studies, and qualitative studies (Aromataris et al., 2015). A ‘yes’ answer was scored as 1 point, ‘unclear’, or ‘no’ answers were scored as 0 points; higher score indicating higher quality. Methodological quality of a study was classified as high (≥2/3 of maximum score), moderate (1/3 < score < 2/3) and low (≤1/3) for each tool. One reviewer (ZC) appraised the quality of all studies, two reviewers independently reassessed the quality of qualitative (SC) and quantitative (ZZ) studies. Discrepancies were resolved through discussions among three reviewers. Low‐quality studies were not excluded, instead quality of studies was considered for contrasting their results (e.g. low vs. high quality).

### 2.3 Data analysis and synthesis

Data were analysed over two stages. First stage followed a ‘*reflexive thematic analysis*’ process (Braun et al., 2019) to analyse qualitative data, over six reflexive and recursive phases: 1. familiarisation, 2. coding, 3. generating themes, 4. reviewing themes, 5. defining themes and 6. writing‐up. Findings and characteristics of each study was extracted into a spreadsheet (phase one), which was analysed line‐by‐line, any statement addressing patients' engagement or their experience of participating in their own care was coded (phase two). Themes were generated by identifying patterns in codes (phase three), then was reviewed and compared within and across the included studies (phase four), defined in phase five each theme, and written up (phase six). This approach was deemed most appropriate for analysing the qualitative data in this review because it examined people's experiences, views and perceptions, and representations of a given phenomenon (Braun et al., 2019). These themes laid the foundation for the second stage of analysis.

Stage two, following the JBI guidelines, quantitative data were qualitised using ‘*deductive thematic analysis*’ (Braun & Clarke, 2006, p.83), matched them to existing themes, then transformed into textual descriptions or narrative interpretation of the quantitative results (Stern et al., 2020). New themes could emerge at this stage, but none did. ZC, ZZ, SC and WS developed the data extraction sheet, ZC extracted the data, ZC, ZZ and SC took part theme generation and data analysis.

The reporting of this review followed The Preferred Reporting Items for Systematic reviews and Meta‐Analyses PRISMA 2020 (Appendix S2) (Page et al., 2021).

## 3 FINDINGS

**FIGURE 1:**
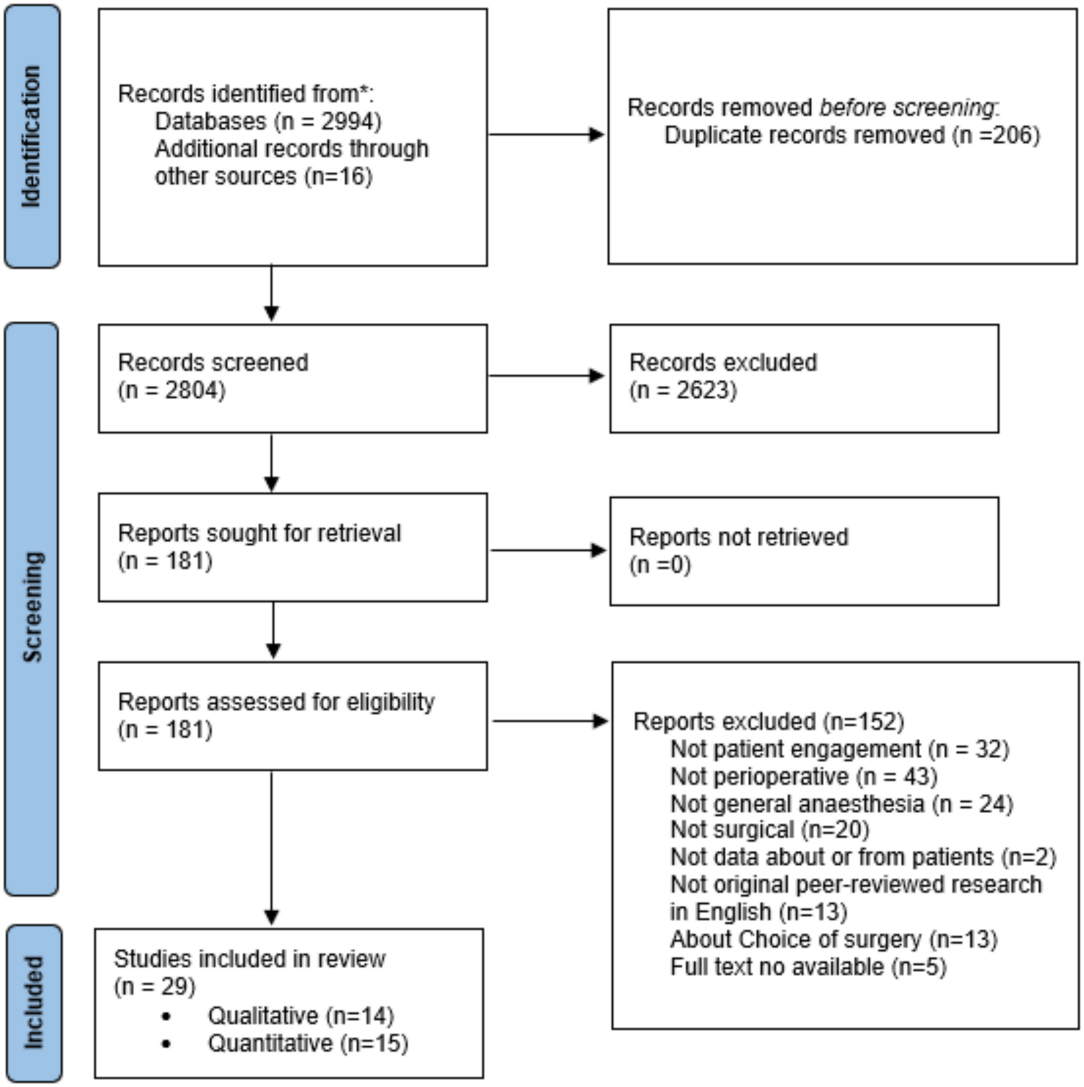
PRISMA 20220 flowchart of study selection. [Colour figure can be viewed at wileyonlinelibrary.com]

The process of study selection is shown in Figure 1. In total, 14 qualitative, and 15 quantitative studies, including two randomised control trials, five quasi‐experimental, and eight cross‐sectional studies were included.

Six studies were conducted in the United States, three in Sweden, three in Australia, two in Norway, two in Denmark and four in other European countries (Austria, Switzerland, Netherlands, Finland and one from each country). Two studies were conducted across multiple European countries (one in Czech Republic, Cyprus, Finland, Greece and Hungary; and another across Finland, Spain, Greece, Germany and Scotland). The remaining were conducted in Asia: two in Taiwan, one in China and one in Thailand. The sample sizes in each study varied from 10 to 2275 (total sample *n* = 6289); and all studies were conducted in medical centres or hospitals. Included studies and their characteristics are presented in Table 1.

**TABLE 1:** Characteristics of included studies.

Author	Place	Study design	Sample size	Gender	Age	Setting	Research question/aim
Aasa et al. (2013)	Sweden	Qualitative – interview	Patients (*n* = 12)	Both	Adult patients 46–73	Surgical clinic of a county hospital in Southern Sweden	The primary aim of this study was to identify and describe patients' experiences of the ERAS‐conversation with nurses. The secondary aim was to identify patients' participation in their own care
Andersson et al. (2015)	Sweden	Qualitative – interview imbedded within control	Patients (*n* = 18)	Both	Adult patients 46–82	Public Hospital	To explore patients' perceptions of preoperative information about pain treatment as well as its importance for the way pain was managed in the postoperative phase
Bhardwaj et al. (2012)	UK	Qualitative – Interview	Patients (*n* = 10)	Both	Adult patients 27–65	Neurosurgical ward within a large, regional hospital	To explore patients' beliefs and perceptions regarding perioperative urinary catheterisation, and Relate patients' beliefs to current and future practice
Castellanos et al. (2018)	United States	Qualitative – interview	Patients (*n* = 38)	Both	Adult patients 18–70	A private hospital and a safety net hospital in southwest USA	To examine the hypothetical signalling game against the communication patterns that were noted in the interviews to understand what conditions and behaviours may be optimised to enhance engagement
Conradsen et al. (2016)	Norway	Qualitative – interview	Patients (*n* = 11)	Unclear	Adult patients 56–82	Surgical department at a hospital in a town in a rural area on the west coast of Norway	To investigate how patient education in a surgical department was experienced by patients who had undergone total knee arthroplasty or total hip arthroplasty
Cook et al. (2013)	United States	Quantitative – Quasi‐experimental	Patients (*n* = 185)	Both	Adult patients 52–85	Hospital	To test whether an e‐health platform could support the delivery and acquisition of patient reported outcomes (PROs) during hospitalisation after cardiac surgery
Hanucharurnkui and Vinya‐nguag (1991)	Thailand	Quantitative – RCT	Patients (*n* = 40) Intervention (*n* = 20) Control (*n* = 20)	Both	Adult patient 22–60	University Hospital, Chiang Mai	To test the effects of promoting patient participation in self‐care, on postoperative recovery from surgery and satisfaction with care
Heggland and Hausken (2013)	Norway	Qualitative – interviews	Patients (*n* = 7) Health Professional (*n* = 11)		Adult patients 25+	Stavanger University Hospital, Norway	To clarify patient participation by specifying kinds of information flows between healthcare professionals and patients in four models such as the paternalistic, shared, informed and no paternalistic models
Henselmans et al. (2012)	Netherlands	Qualitative – Interviews	Patients (*n* = 20)	Both	Adult patients 42–77	Academic Medical Center (AMC) in Amsterdam	To examine (1) the content and type of patients' information needs and (2) patient perceived facilitators and barriers to patient participation
Hou et al. (2014)	China	Quantitative – Cross‐sectional	Patients (*n* = 113)	Both	Adult patient 22–91	General surgical ward in two of the upper first‐class hospitals in Beijing	To compare preferred participation roles to actual roles and the degree of their concordance during treatment decision‐making in the sample of colorectal cancer patients just after surgical treatment
Ivarsson et al. (2005)	Sweden	Quantitative – Quasi‐experimental	Patients (*n* = 434) Intervention (*n* = 220) Control (*n* = 214)	Both	Adult patients 25–83	Cardiothoracic surgical centre in Sweden	The aim was to describe patients' experiences of information regarding possible complications related to cardiac surgery, both before and after the operation
Jørgensen and Fridlund (2016)	Denmark	Qualitative – Grounded Theory	Patients (*n* = 14)	Both	Adult patients 52–78	Total hip arthroplasty program at Regional Hospital Silkeborg, Denmark	The aim of this study was to generate a theory conceptualising and explaining behavioural processes involved in coping in order to identify the predominant coping types and coping type‐specific features
Kaptain et al. (2017)	Denmark	Qualitative – Interviews	Patients (*n* = 15)	Both	Adult patients 20–81	Orthopaedic unit at Aarhus University Hospital	To explore how patients undergoing spine surgery participated in postoperative pain assessment in a recovery unit
Lane‐Carlson and Kumar (2012)	United States	Qualitative ‐Interviews	Patients (*n* = 24)	Both	Adult patients (age range unclear)	Kaiser Permanente Downey Medical Center	To engage patients in managing their health care especially in relation to a total joint replacement (TJR). With the aging of the American population and the advent of new technology, there is an increase in TJRs. As the pendulum swings from evidence‐based medicine to patient‐centred medicine, presurgical education is preparing patients for their surgical experience. Most research studies on such education are quantitative in nature, preventing patients' voices from being heard
Lo et al. (2010)	Taiwan	quantitative – Quasi‐experimental	Patients (*n* = 102) Intervention (*n* = 46) Control (*n* = 56)	Both	Adult patients (age range unclear)	A surgical unit in a 900‐bed medical centre in Taiwan	The aim of the study was to evaluate the effectiveness of a multimedia education programme in relation to stoma knowledge, self‐care attitudes and behaviour with patients with a stoma in the postoperative period
Mahler and Kulik (1990)	United States	Quantitative – Cross sectional –	Patients (*n* = 75)	Male	Adult patients 38–69	San Diego Veterans Administration Hospital	To determine whether and how patients' preoperative perceptions of control over their recovery might actually facilitate recovery from coronary artery bypass surgery
Mata et al. (2017)	Canada	Quantitative – cross sesctional	Patients (*n* = 223)	Both	Adult patients (age range unclear)	McGill University Health Centre, Montreal General Hospital site	To estimate the extent to which patient, procedural and organisational factors predict adherence to postoperative ERP elements in laparoscopic colorectal surgery
McTier et al. (2013)	Australia	Qualitative – Interviews + naturalistic observations + focus groups	Patients (*n* = 130) – pre‐admission and pre‐discharge patient interviews (*n* = 98), – naturalistic observations (*n* = 48) Nurses focus group interviews (*n* = 2)	Both	Adult patients 25–87	Cardiothoracic ward of a major metropolitan, tertiary referral hospital in Melbourne, Australia	To explore patient participation in the context of medication management during a hospital admission for a cardiac surgical intervention of patients with cardiovascular disease
McTier et al. (2016)	Australia	Qualitative – Interviews + naturalistic observations + focus groups	Patients interviews: preadmission (*n* = 130) pre‐discharge (*n* = 98), naturalistic observations (*n* = 48) nursing focus groups (*n* = 2).	Both	Adult patients 65+	Cardiothoracic ward of a major metropolitan, tertiary referral hospital in Melbourne, Australia	To explore patients' ability and willingness to participate in pulmonary interventions and nurses' facilitation of pulmonary interventions
Meredith (1993)	UK	Qualitative – Interviews and non‐participant observation	Non‐participant observation: Patients (*n* = 10) Interviews: Patients (*n* = 30) Surgeons (*n* = 14)	Both	Adult patients ‘with as broad an age range as possible’	General elective surgery at six hospital sites	The objective in this investigation was to explore the entire experience of one surgical specialism, general surgery, from GP referral to the outpatients consultation to point of discharge, with special reference to patients' attitudes to and evaluations of communication, information and consent
Ong et al. (2009)	United States	Quantitative – Quasi‐experimental	Patients (*n* = 15) Nurses (*n* = 18)	Both	Adult patients (age range unclear)	University of California	To determine the effects of developing and implementing an innovative preoperative instructional DVD on patients' level of knowledge, preparedness and perceived ability to participate in postoperative care activities at a university‐affiliated public medical centre
Page et al. (2021)	Australia	Quantitative – RCT	Patients (*n* = 96) Intervention (*n* = 53) Control (*n* = 39)	Both	Adult patients (age range unclear)	Ipswich Hospital NHS Trust	To determine whether the passport would help people with diabetes undergoing elective surgery feel better informed and more involved in their diabetes care at various stages of the perioperative process
Papastavrou et al. (2015)	Czech Republic, Cyprus, Finland, Greece and Hungary	Quantitative – Cross‐sectional	Patients (*n* = 1315) Nurses (*n* = 960)	Both	Adult patients (age range unclear) Nurses (age range unclear)	Hospitals in five European countries	To analyse and compare patients' and nurses' perceptions of patients' decisional control over their own care
Timonen and Sihvonen (2000)	Finland	Quantitative – Cross‐sectional	Patients (*n* = 74) Nurses (*n* = 118)	Both	Adult patients aged 18 years and over Nurses aged 24–75	Abdominal and orthopaedic wards at six hospitals in Helsinki, Finland	The aim of this study was to compare nurses' and patients' opinions of the purpose of bedside reports, patient participation in bedside reporting sessions, and factors that promote or prevent their participation
Trummer et al. (2006)	Austria	Quantitative – Quasi‐experimental	Patients (*n* = 199) Control *n* = 100 Intervention *n* = 99	Both	Adult patients aged 18 years and over	Cardiac surgery department at an Austrian University Hospital	The study examined two research questions: first, whether a training in skills of patient centred communication for professionals and improvements in patient information talk schemes have effects on objective and subjective clinical outcome parameters. Second, how can such effects be explained, as unspecific (a Hawthorne effect), or as specifically related either to the informational or to the emotional quality (relational aspect) of provider–patient communication
Uldry et al. (2013)	Switzerland	Quantitative – Cross‐sectional	Patients (*n* = 253)	Both	Adult patients aged 18 years and over	Department of Visceral Surgery, University Hospital of Lausanne, Switzerland	This study aimed at assessing patients' preferences on different aspects of decision‐making during treatment and potential complications, as well as the amount and type of preoperative information wanted before visceral surgery
Valimaki et al. (2004)	Finland, Spain, Greece, Germany, Scotland (UK)	Quantitative – Cross‐sectional	Patients (*n* = 1454) Finland (*n* = 464) Spain (*n* = 173) Greece (*n* = 275) Germany (*n* = 254) Scotland UK (*n* = 288)	Both	Adult patients aged 18 years and over	Surgical department of five hospitals in five European countries	To analyse the effects of informational support, desire for behavioural involvement in health decision‐making (behavioural involvement), opportunities to make decisions, and independence on subjective health status in surgical patients. A theoretical model of self‐determination was applied and tested
Wilcox et al. (2016)	United States	Qualitative – Interviews	Phase 1 – patients (*n* = 20) Phase 2 – patients (*n* = 12), pharmacist (*n* = 5)	Both	Adult patients (age range unclear)	Postoperative cardiothoracic surgery step‐down unit at Columbia University Medical Center at New York‐Presbyterian Hospital (NYP)	To explore the design and usefulness of patient‐facing tools supporting inpatient medication management and tracking
Yeh et al. (2005)	Taiwan	Quantitative – Cross‐sectional	Patients (*n* = 66) intervention (*n* = 33) control (*n* = 33)	Both	Adult patients (age range unclear)	Seven orthopaedic or surgical departments in a 4000‐bed medical teaching hospital in northern Taiwan	To explore effects of multimedia CD with printed nursing guides in patient education on the improvement of self‐efficacy, functional activity and length of hospitalisation in patients with hip replacement

### 3.1 Quality appraisal

**FIGURE 2:**
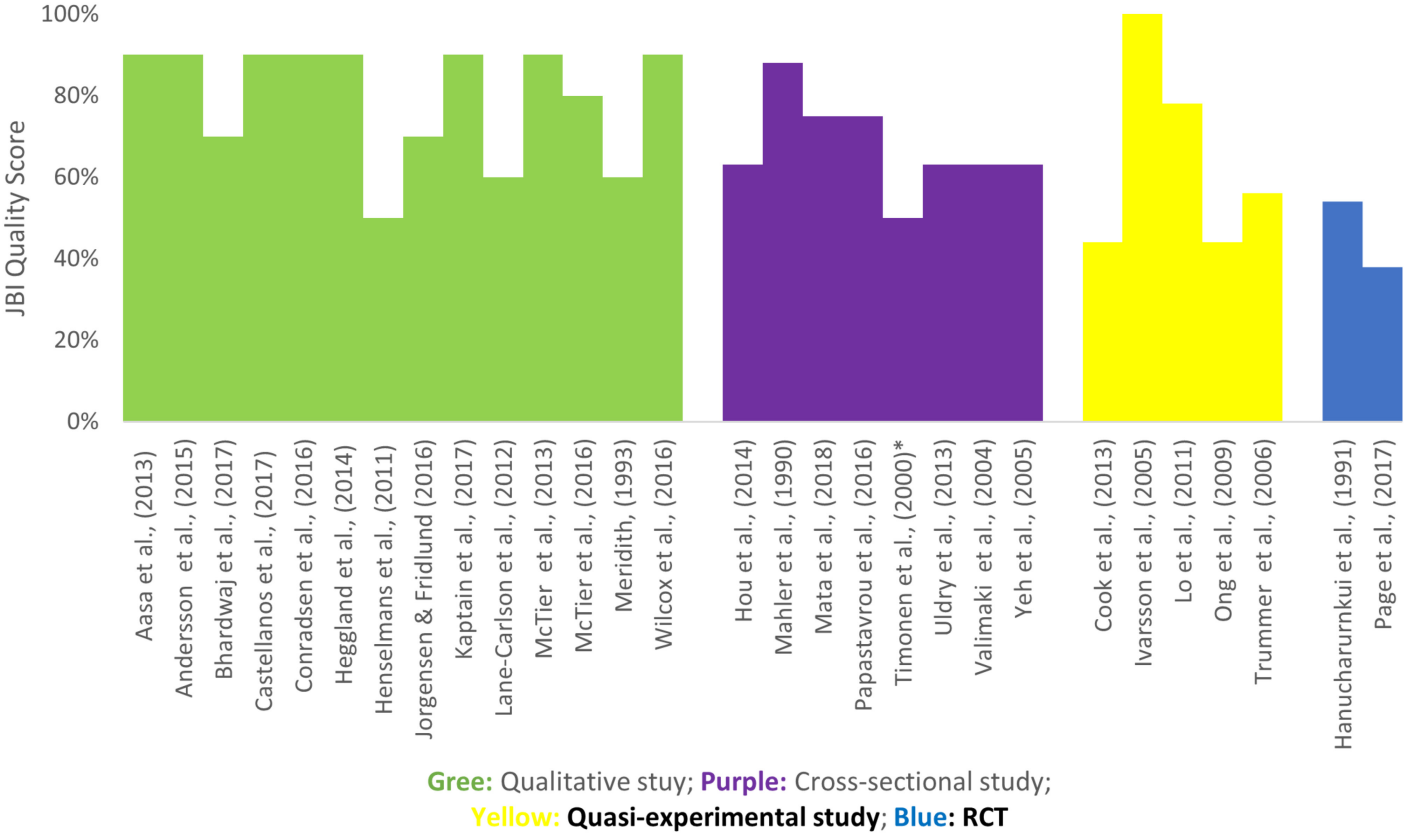
Quality appraisal of the included studies. [Colour figure can be viewed at wileyonlinelibrary.com]

Figure 2 summarise the quality appraisal for each study. Three of 14 qualitative studies had a moderate quality, remaining 11 were high quality. Of eight cross‐sectional studies, five were moderate and three were high quality. Of five quasi‐experimental studies, three were moderate and two were high quality. The two randomised control trials were both of moderate quality.

### 3.2 Synthesis of results

Conceptualisations of surgical patient engagement and operationalisations of surgical patient engagement are presented in this section.

#### 3.2.1 Defining surgical patient engagement – Conceptualisations

Not all included articles offered an explicit definition of patient engagement. Table 2 outlines the focus and working definition for patient engagement (if applicable).

**TABLE 2:** Conceptualisations of surgical patient engagement.

Paper	Focus of study	Working definition	Associations
Aasa et al. (2013)	Preoperative information	‘*an established relationship between a nurse and a patient in which there is a transfer of some power and control to the patient* (*Sahlsten et al., 2008*)’	The patient–provider relationship Perceived control
Andersson et al. (2015)	Provision of preoperative information	–	–
Bhardwaj et al. (2012)	Patients' perceptions and provision of information	–	–
Castellanos et al. (2018)	Communication	‘*an engaged patient is generally thought of as someone willing to reach out to his caregivers with questions or concerns and is intent on understanding their condition to actively benefit the outcome*’	Actions
Conradsen et al. (2016)	Provision of information and education programme ahead of orthopaedic surgery	–	–
Cook et al. (2013)	Provision of information via an eHealth platform	‘*combining ‘patient activation’ and engagement and begins with providing information to patients in ways that enable them to actively participate in their care*’	Perceived control or power by the patient Providing information Perceived control
Hanucharurnkui and Vinya‐nguag (1991)	Provision of information and communication	*‘a process of perception and communication between person and person*, *represented by verbal and nonverbal behaviours that are goal‐directed*	Communication
Heggland and Hausken (2013)	Provision of information and patient participation in decision‐making	‘*Patient participation represents patients taking part in decision‐making processes and influences the decision‐making process and the service he receives*’	Actions Communication Decision‐making
Henselmans et al. (2012)	Postoperative information	–	–
Hou et al. (2014)	Decision‐making	–	–
Ivarsson et al. (2005)	Provision of information	–	–
Jørgensen and Fridlund (2016)	Decision‐making	–	–
Kaptain et al. (2017)	Patient participation in pain management via Communication	–	–
Lane‐Carlson and Kumar (2012)	Preoperative educational programs	‘*When educational needs are met before surgery*, *patients are more engaged in their medical care and sense an improvement in their quality of life*’	Providing information
Lo et al. (2010)	Decision‐making	–	–
Mahler and Kulik (1990)	Provision of information, decisional control and behavioural involvement in health care	‘*Perceived control and desire for health care involvement*’	Perceived control
Mata et al. (2017)	Adherence to postoperative interventions (Action‐taking)	–	–
McTier et al. (2013)	Patient participation in medication management (communication and provision of information)	‘*… active*, *involved and informed participants in the health‐care team*’	Actions
McTier et al. (2016)	Patient participation in postoperative pulmonary interventions (action‐taking)	–	Actions
Meredith (1993)	Patient participation in decision‐making	–	–
Ong et al. (2009)	Provision of information via DVD	–	–
Page et al. (2021)	Provision of information in the form of a diabetes ‘passport’	‘*…empowerment as being a patient‐centred*, *collaborative approach tailored to match the fundamental realities of diabetes care* (*Funnell & Anderson, 2004*)’	The patient–provider relationship
Papastavrou et al. (2015)	Patient decisional control over care (decision‐making)	‘*an established relationship between nurse and patient*, *a surrendering of some power or control by the nurse*, *shared information and knowledge*, *and active engagement together in intellectual and/or physical activities*’	The patient–provider relationship Perceived control
Timonen and Sihvonen (2000)	Communication	–	–
Trummer et al. (2006)	Communication	–	–
Uldry et al. (2013)	Provision of information and participation in decision‐making	–	–
Valimaki et al. (2004)	Provision of information and participation in decision‐making	–	–
Wilcox et al. (2016)	Provision of information and communication	‘*we looked to conceptualizations of ‘activation’ and ‘participation’ – each originating in the Chronic Care Model* (*Bodenheimer et al., 2003*)’	Actions The patient–provider relationshipProviding information
Yeh et al. (2005)	Provision of Information	*–*	–

Only 11 (38%) of the included studies referred to an explicit definition of patient engagement. Of the 11, two studies offered a working definition of their own (McTier et al., 2013, 2016) and nine referenced a definition from existing research.

Five of the 11 studies focussed on *patient actions* (McTier et al., 2013, 2016; Wilcox et al., 2016). Four explored *perceived control or power by the patient* (Aasa et al., 2013; Cook et al., 2013; Mahler & Kulik, 1990; Papastavrou et al., 2015). Three definitions address *providing information* to patients to promote engagement (Cook et al., 2013; Lane‐Carlson & Kumar, 2012; Wilcox et al., 2016). Four definitions focussed on the *patient–provider relationship* (Aasa et al., 2013; Lane‐Carlson & Kumar, 2012; Wilcox et al., 2016). Two definitions focussed on communication (Hanucharurnkui & Vinya‐nguag, 1991; Heggland & Hausken, 2013). While only a single definition focussed on decision‐making (Heggland & Hausken, 2013).

Other related concepts that appear in working definitions include patient participation (Cook et al., 2013; Heggland & Hausken, 2013; McTier et al., 2013, 2016; Papastavrou et al., 2015; Wilcox et al., 2016), patient activation (Cook et al., 2013; Wilcox et al., 2016) and patient empowerment (Aasa et al., 2013; Cook et al., 2013; Lane‐Carlson & Kumar, 2012). In sum, surgical patient engagement during the perioperative period has been conceptualised as provision of information (Cook et al., 2013; Lane‐Carlson & Kumar, 2012; Wilcox et al., 2016), communication (Castellanos et al., 2018; Hanucharurnkui & Vinya‐nguag, 1991; Heggland & Hausken, 2013), decision‐making (Heggland & Hausken, 2013), patients' actions (McTier et al., 2013, 2016; Wilcox et al., 2016), as well as perceived control by the patient (Aasa et al., 2013; Cook et al., 2013; Mahler & Kulik, 1990; Papastavrou et al., 2015) and patient–provider relationship (Aasa et al., 2013; Lane‐Carlson & Kumar, 2012; Papastavrou et al., 2015; Wilcox et al., 2016).

#### 3.2.2 Defining surgical patient engagement—Operationalisation

**FIGURE 3:**
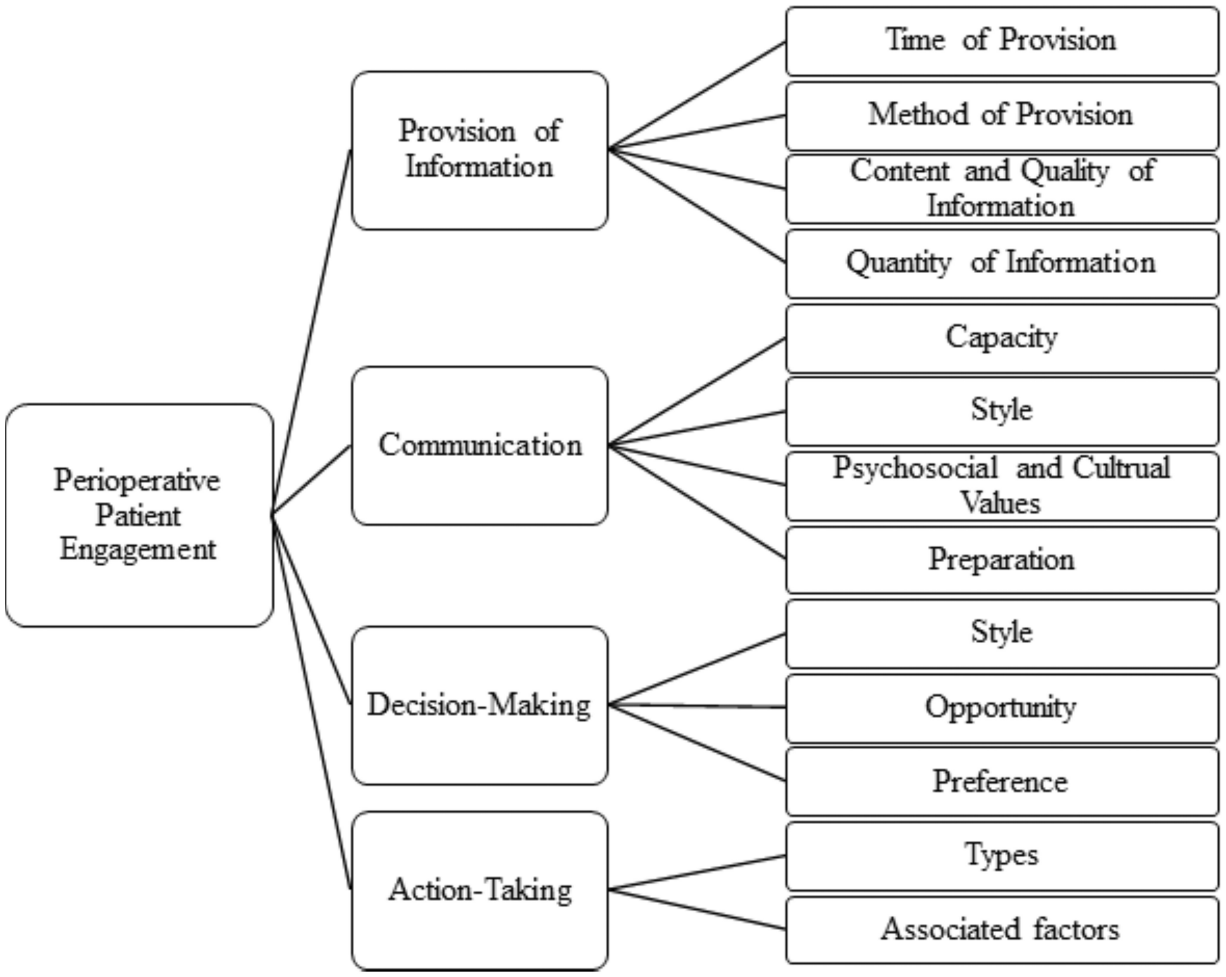
A summary of themes and sub‐themes.

Four major themes associated with patient engagement during the perioperative period were identified. These themes were 1. *Provision of Information*, 2. *Communication*, 3. *Decision‐Making*, and 4. *Action‐taking*. These four themes also appear above in *Conceptualisation*; however, two themes of *patient interactions* and *perceived control* do not appear as standalone themes in this section. The theme *Patient interactions* was dissolved under the theme of communication, and *perceived control* likewise was dissolved under the themes of communication, decision‐making and action‐taking. Below, each of the four themes in this section are explored, detailing working definitions of these terms within the scope of this review and their associated subthemes. Table 3 shows the themes originating from each included study. Figure 3 outlines the four themes and subthemes.

**TABLE 3:** Association of each study with each theme.

Study	Provision of information	Communication	Decision‐making	Action‐taking
Aasa et al. (2013)	○			○
Andersson et al. (2015)	○		□	
Bhardwaj et al. (2012)	○		○	
Castellanos et al. (2018)		○		
Conradsen et al. (2016)	○			
Cook et al. (2013)	□			
Hanucharurnkui and Vinya‐nguag (1991)	□	□		
Heggland and Hausken (2013)		○	○	
Henselmans et al. (2012)		○		
Hou et al. (2014)			□	
Ivarsson et al. (2005)	□			
Jørgensen and Fridlund (2016)				○
Kaptain et al. (2017)		○		
Lane‐Carlson and Kumar (2012)	○			
Lo et al. (2010)	□			
Mahler and Kulik (1990)	○		○	
Mata et al. (2017)				□
McTier et al. (2013)	○	○	○	
McTier et al. (2016)	○			○
Meredith (1993)			○	○
Ong et al. (2009)	□			
Page et al. (2021)	□			
Papastavrou et al. (2015)	□		□	
Timonen and Sihvonen (2000)		□		
Trummer et al. (2006)		□		
Uldry et al. (2013)			□	
Valimaki et al. (2004)	□		□	
Wilcox et al. (2016)	○	○		
Yeh et al. (2005)	□			

*Note*: ○ = Qualitative research; □ = Quantitative.

#### 3.2.3 Provision of information

Provision of information was the most prominent theme, with 80 codes derived from 15 studies (51.7% of all papers), including six quantitative and nine qualitative studies.

In this review, provision of information was viewed as any information that is provided to patients by healthcare provider (clinicians) or healthcare organisation (hospital). It is one‐way communication, from clinician/hospital to patient. Three key components were identified: (1) time of provision, (2) method of provision, and (3) quality and quantity of information.

*Time of provision* refers to the time period during a patient's surgical journey when information was provided. It was recommended that information about the surgery, hospital stay and recovery programme should be communicated to patients one week prior to surgery, and followed up at discharge and postdischarge (Aasa et al., 2013). Overall, information needs to be provided multiple times at different intervals during the whole patient surgical journey (Aasa et al., 2013). In addition, information should be relayed to patients, in various *methods*, including verbal, written, digital or pictographic and multimedia. Written information is deemed to be accessible and acting as a point of reference and a reminder for patients to refer to when needed, whereas verbal information is consistently shown to be difficult for information retention and recall. (Aasa et al., 2013; Andersson et al., 2015; Conradsen et al., 2016; Lo et al., 2010; Ong et al., 2009; Wilcox et al., 2016). A combination of different methods is recommended. When information is provided in written or digital form, then followed by verbal provision, it not only reinforces the provided information, but also increases patient's self‐efficacy to formulate questions and engage in communication with their clinicians (Andersson et al., 2015; Lane‐Carlson & Kumar, 2012).

*Quality and quantity of information* is another important consideration when providing information to patients. Large volumes of information delivered over a short period caused difficulties for patients to retain the information, or patients selectively read only some parts (Aasa et al., 2013). Providing sufficient information was considered important. Ivarsson et al. (2005) compared ‘standard information’ with ‘extended and comprehensive information’. Patients who received extended perioperative information were more satisfied (written extended vs standard: 96.1% vs 64.8% *p* < .001; verbal: 79.2% vs 69.7% *p* < .001) and postoperative information (written: 90.7% vs 70.8% *p* < .01; verbal: 80.5% vs 71.9% *p* < .01).

In sum, while information provided should sufficiently meet the patients' needs, it should be provided in easily retainable volumes, over various time points. Patients' understanding should also be assessed (Aasa et al., 2013).

#### 3.2.4 Communication

Communication was discussed in six studies (20.6%) (Castellanos et al., 2018; Henselmans et al., 2012; Kaptain et al., 2017; McTier et al., 2013; Timonen & Sihvonen, 2000; Trummer et al., 2006; Wilcox et al., 2016) It was defined as the exchange of information between patients and their clinicians. Unlike provision of information, communication involves two‐way exchange of information (verbal and non‐verbal) and includes communications with the family or carers. Communication is beyond exchange of information, it is more nuanced and includes emotional and psychosocial attributes. This analysis identified four key components: (1) patient's capacity to communicate, (2) communication skills, (3) patient‐preparation and (4) psychosocial and cultural values.

*Patient's capacity to communicate* can be easily impacted by their physical and/or mental health, such as pain and medication use, including general anaesthetics, analgesics or other medication. Kaptain et al. (2017) state that some of the patients had difficulty verbalising or describing their pain, largely due to the severity of their pain or side effects of medications.

*Communication skills* of patients and clinicians could be easily overlooked by clinicians in clinical practice. Timonen and Sihvonen (2000) study that examined patient compared with nurses' perception of bedside handover found that 83% of the nurses believed that both patient and nurses took part in the conversation; however, 48% of patients disagreed with this statement. The differences in opinion were influenced by whether patients were encouraged to take part in communication during bedside reporting. About 91% of nurses felt that patients were encouraged to participate, whereas only 50% of patients agreed with this statement. To facilitate a dialogue and participate in communication, communication skills of both clinicians and patients are essential.

*Patient‐preparation* may be a direct result of provision of information. Patients who are not effectively prepared or informed prior to consultation or surgery may not engage in communication, as they may either not understand the topic, or be able to ask relevant questions. In Henselmans et al. (2012), patients recommended a written question prompt sheet or a website including example question. Patients' *psychosocial characteristics* may influence their engagement in communication with their clinicians. The study by Henselmans et al. (2012) found that a patient's personality, beliefs and prior surgical experience, as well as family support and clinician's personality, can impact their communication.

Communication between patients and clinicians is a complex dynamic, not only impacted by the skills and capacity of both clinicians and patients to communicate, but also by patients' preparedness and their psychosocial features.

#### 3.2.5 Decision‐making

Decision‐making theme was derived from 49 codes, appearing in six studies (20.7%) (Heggland & Hausken, 2013; Hou et al., 2014; McTier et al., 2016; Papastavrou et al., 2015; Uldry et al., 2013; Valimaki et al., 2004). Decision‐making is the process of identifying choices based on values, preferences and beliefs of the decision‐maker. In this review, decision‐making is a process in which clinicians and patients make decisions regarding patients' care. Three key components include: (1) style, (2) opportunity and (3) demographics.

As illustrated in Table 4, there are two *styles* with one from clinician's point of view (Heggland & Hausken, 2013) and the other from patients (Hou et al., 2014).

**TABLE 4:** Various decisions‐making models.

Decision‐making models [22]		Decision‐making models [42]	
Paternalistic	Positions the clinician above and patients as passive recipients of care	Passive	Patients prefer to leave all decisions regarding treatment to clinician
Shared	Emphasises the exchange between clinicians and patients	Shared	Patients prefer to share responsibility for treatment decisions with clinician
Informed	Clinician provides the necessary information to patients to make own treatment decisions	Active	Patients prefer to make the final decision about any treatment after seriously considering clinician's opinion
non‐paternalistic	Clinicians treat patients as principals who delegate authority to clinicians to take action	Family plenipotentiary	Patients have no involvement in the decision‐making process and a family member has full power

*Whether decision is made also depends on opportunity*. Patients may not be aware that they have a choice, or they may perceive a lack of opportunity to be involved in decision‐making. This clearly comes up in the Bhardwaj et al.'s (2012) study, where patients perceived that there were few choices during the treatment process.

*Demographics* such as age, gender and education impact on capacity and willingness to make decisions. Uldry et al. (2013) found that older patients, women and patients with no education or with a superior education had less desire to be involved in the medical decisions than younger patients (on average − 0.20 points every 10 years; *p* = .003), males (*p* = .04) or those with an average educational level (average score 4.11 and 3.82 vs 4.62; *p* = .015), respectively.

In sum, clinicians and patients' preferences for involvement in decision‐making and their perception of available opportunities may differ.

#### 3.2.6 Action‐taking

Taking action refers to any patient behaviour that aids their surgical recovery or portrays a sense of autonomy in their surgical journey. This definition emphasises the importance of self‐efficacy, autonomy, or at the very least, adherence and compliance. Research on this concept is limited. This theme was derived from 33 codes in only four (13.8%) studies. The two key components are: (1) types of action‐taking and (2) contributing factors.

Dependent action results from a need or desire to follow directions. Aasa et al. (2013) state that many of the patients in their study understood their role as a patient and were aware of their responsibilities. They did what they were instructed to do despite pain, poor appetite and or other discomfort. Autonomous action‐taking behaviour refers to patients who have the confidence and self‐efficacy to take charge of their own recovery. These patients often require minimal instructions or feedback and will seek advice when and if they require it. For instance, they may take steps to arrange appointments with their GP after the consultation with their surgeon (Meredith, 1993). This action was not prompted by the advice of the clinicians, rather it was a proactive approach on the patient's part.

Jørgensen and Fridlund (2016) identified four types of ‘coping behaviour’ of surgical patients that are linked to types of decision‐marking: exceeding the boundaries of capability, protecting the boundaries of capability, challenging the boundaries of capability and accepting the boundaries of capability (Appendix S3). They then further identified whether each type was a match or mismatch to their fast‐track programme, an accelerated joint recovery programme for patient undergoing total hip arthroplasty.

What could contribute to different styles of action taking is not well‐researched. Patient's physical health status during the postoperative period may impact their participation and actions. Mata et al. (2017) reported that the independent predictors of lower mean adherence during the postoperative phase was PONV (15.4% reduction, 95% CI −26 to −4.6, *p* = .006) and late arrival at the ward after 6 p.m. (9% reduction, 95% CI −16 to −2%, *p* = .007). Patients with PONV mobilised from bed were less likely to ingest protein supplement or solid food as recommended in their recovery plan.

To summarise, patient's engagement in action‐taking may be autonomous or dependent subject to the advice and feedback of clinicians. Both could result in successful with guidance and encouragement.

### 3.3 Interactions and interrelationships among themes

**FIGURE 4:**
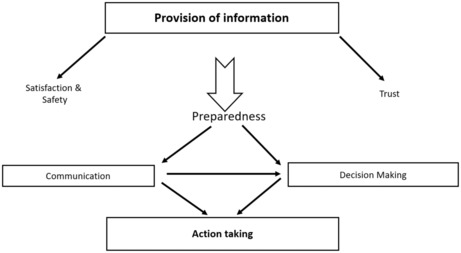
Concept map of themes of patient engagement during perioperative care.

All four themes of surgical patient engagement are interrelated (Figure 4). Provision of information may lead to patients feeling prepared and safe (Aasa et al., 2013; Conradsen et al., 2016), and being able to make informed choices and take part in decision‐making (Valimaki et al., 2004). Provision of information may be associated with patient satisfaction. For instance, in a study by Ivarsson et al. (2005), 337 cardiac surgery participants were surveyed for their experiences related to pre‐operative information about possible complications of cardiac surgery. The intervention group received extended pre‐operative information about possible complications, whereas control group did not. The intervention group was significantly (*p* < .01) more satisfied with written and oral information provided about common complications. Similarly, Hanucharurnkui and Vinya‐nguag (1991) looked at effects of information provision on patient participation. The experimental group in the study reported significantly higher scores on patient satisfaction (M = 164.75, SD = 9.52) than those in the control group (M = 147.25, SD = 11.45), *t* (38) = 5.25, *p* < .001. One other study suggests provision of information is also associated with patients' involvement in decision‐making (Valimaki et al., 2004). This study investigated informational support provided to patients and its impacts on patients' behavioural involvement and decision‐making and they reported ‘*the extent to which patients received information about their own care from health care professionals strongly correlated overall with the opportunity to make decisions related to everyday care* (*r  = .76*, *p < .001*)’ (p.308). Furthermore, provision of explicit, honest and consistent information can lead to feelings of trust, whereby the patient perceives the information as reliable and trusts in the process and the ability of clinicians to provide care. The patient's trust can be challenged when there is incongruency in the information provided throughout their surgical journey.

Overall, it is important to acknowledge that surgical patient engagement is multifaceted; and should be approached holistically and viewed as a journey.

## 4 DISCUSSION

This review finds that most (*n* = 18, ~62%) of the included studies did not provide a definition of patient engagement in their research. The absence of a working definition in so many papers support our augment of a conceptual void in the research. This void is discussed in more detail below. The second key focus was on the operationalisation of patient engagement. In sum, four key themes were found, in order of prevalence: (1) provision of information (*n* = 15, 51.7%), (2) communication (*n* = 7, 24.1%), (3) decision‐making (*n* = 7, 24.1%) and (4) action‐taking (*n* = 4, 13.8%). The four themes are interrelated and interconnected to form a holistic picture of surgical patient engagement. Below, implications of these findings and their connections to broader research literature are discussed.

### 4.1 Contextuality of setting

Provision of care differs across healthcare settings. As such, the healthcare setting is also pertinent to the concept of patient engagement (Davis et al., 2007; Liang et al., 2017), which inspired the rationale for conducting this review. Patient journey in primary/secondary care differs from that in a tertiary healthcare setting. Perioperative patient engagement follows a patient's journey from the preoperative stage, to postoperative and discharge. Conversely, engagement in other clinical settings may not resemble a journey, but rather a specific time point. Furthermore, the relationship between patients and clinicians may differ across healthcare settings. For instance, in primary/secondary care, clinicians could follow a patient's development and medical history for several years. This continuity of care provides opportunities for building rapport, as patients may see the same clinicians over a long period. Conversely, in perioperative settings, patients are required to interact with different health professionals over a day or a week; hence, rapport may not be established as easily in such settings. Additionally, surgical patients have less autonomy in comparison with primary/secondary care settings, purely due to the care needs of the patient. This lack of autonomy, followed by unavoidable procedures such as changing into hospital gowns, surrendering belongings and further relinquishing all control by accepting to undergo general anaesthesia, may be conducive to patients feeling disempowered in surgical settings (Faulkner, 2001). Furthermore, as mentioned earlier, surgeries that are conducted under general anaesthesia are often associated with longer hospital stay (Neuman et al., 2014), longer periods of recovery (Gabriel et al., 2017; Ganter et al., 2014) and postoperative complications such as postoperative pain (Ganter et al., 2014) and PONV (Apfel et al., 1999). These challenges influence patient engagement.

Our review examines patient engagement in direct health care and how it can be improved. Graffigna and Barello (2022) suggest considering patient engagement at three levels: micro (individual patient strategies), meso (engaging patients in healthcare service design and technology assessment) and macro (patient involvement in policy). Our review specifically focusses on the microlevel, or individual patient strategies for self‐care and shared decision‐making.

In summary, it is crucial to be cognisant of the importance of contextuality when undertaking patient engagement research in perioperative settings and beyond.

### 4.2 Interpretations of findings: Conceptualisation of patient engagement

This review had only two studies (6.9%) that offered a specific working definition of patient engagement in the papers analysed, 11 papers alluded to an explicit definition (37.9%). The widespread absence of a definition of patient engagement in research papers promotes a sense of ambiguity and a theoretical void. Additionally, in cases of interdisciplinary research on topics as broad as patient engagement, a lack of shared understanding of the concept poses a risk to the ambiguity of data collection and research outcomes. Researchers commonly aligned their conceptualisation of patient engagement based on the focus of their research, for instance on provision of information or communication. As such those approaches narrow the broad concept of patient engagement to one or two elements, and the results may miss other elements, such as decision‐making and action‐taking, and/or the intersection or interplay of the four elements identified in this review.

Inconsistency and interchangeability of terms in the research literature including patient engagement, participation, empowerment and activation are problematic. Adding to this complexity, patient activation is often standardised with Hibbard's definition, concepts such as patient engagement, whereas patient participation and patient empowerment do not have one standard agreed‐upon definition. While this review is not necessarily calling for a static, unidimensional definition of patient engagement, it calls for a need for greater awareness of the nuances of discourse terminology and the interchangeable nature of terms. Some of these concerns have been voiced in other studies (Harrington et al., 2020; Manafo et al., 2018).

### 4.3 Interpretations of findings: Operationalisation of patient engagement

The four themes identified in this review, (1) provision of information, (2) communication, (3) decision‐making and (4) action‐taking, highlight different facets of the concept of perioperative patient engagement and should be treated as interconnected and co‐dependent on each other.

A surgical patient's engagement journey starts with provision of information, as it prepares them to communicate, and equips them with the essential information needed for decision‐making and taking actions related to their care. This review has clearly identified that providing information that meets patients' needs, and with more than one method, such as written or digital followed by verbal is preferred by patients. *Time of provision* during a patient's surgical journey, such as before and after surgery, and at discharge, and quantity of information are two understudied areas. There are insufficient data on the optimum time point during the perioperative phase to provide different information to patients. Only one study recommended the best time to provide preoperative information to be one week prior to surgery (Aasa et al., 2013). In terms of quantity of information, none of the studies provided an objective measure of quantity of information or explained what would constitute as too much or too little information. Another important observation was that while sufficient information to the patients was valuable, the nursing staff often opted for providing brief and sometimes insufficient information (McTier et al., 2016). The study, however, did not discuss whether this was due to time constraints, nurses' lack of awareness of patients' needs or other reasons.

Communication is impacted by patients' capacity, psychosocial factors and preparedness to communicate as well as clinicians' communication skills. Patients with insufficient information or a certain style of communication are less likely to formulate questions to pose to clinicians, resulting in missed instructions and an inability to engage in behaviours promoting postoperative recovery. Similar findings were discussed by a study preparing diabetic patients identify questions and priorities before a consultation to improve their care (Grant et al., 2017).

This review identified a significant discrepancy between clinician vs. patient perception of communication. While clinicians commonly believed they were engaging patients in a dialogue, the patient perceived that the information was communicated in a unidirectional style. Nurses were unaware that they were dominating the conversation in up to 80% of the observed conversation (Pettersson et al., 2018; Timonen & Sihvonen, 2000).

Communication style of clinicians can have a considerable impact on the patient's decision‐making, treatment compliance, and satisfaction with care, and ideally to be tailored to patients' preferences and characteristics (Levinson et al., 2013; Rodin et al., 2009). However, a clear recognition of cultural context was lacking in the included studies under this category, which is also observed by Schouten and Meeuwesen (2006), who stated that communication was often not studied considering culture‐related variables, and the effects of cultural variations on communication outcomes were not assessed. Furthermore, a lack of awareness around cultural context has been identified as a barrier to patient–provider communication (Paternotte et al., 2015).

This review identified that active and passive decision‐making styles, which are impacted by the perceived *styles* of decision‐making of both the clinician and the patient (Heggland & Hausken, 2013; Hou et al., 2014). *Opportunity and Preference* go hand in hand and refer to not only choices present to the patient, but also each patient's desire for decision‐making. Our findings suggest that patients may not be aware that they can engage in decision‐making. This observation was shared by Say and Thomson (2003) and Gartner et al. (2019) who state that understanding patients' preferences can improve quality of care. Whereas a lack of ‘choice awareness and a neutral presentation of options’ hinders shared decision‐making. The previous themes of provision of information and communication play an important role in raising awareness and preparing patients to take the opportunity to participate in decisions related to their care, as patients who may prefer to take a passive role in decision‐making, may be encouraged if their informational needs are met. Culture context could determine the preference of decision‐making. The concept of family plenipotentiary (Hou et al., 2014) highlights the central role of the family in the Chinese culture and may not translate into western culture. The Gilbar and Miola (2014) paper also alludes to this point and state that patients from non‐Western backgrounds may have a desire for their family to be involved in decision‐making, this desire may not often be catered for. This is one of the few examples of the importance of cultural context apparent in the concept of patient engagement.

This review found that little attention has been paid to action‐taking with only four studies examining this theme. Action‐taking is closely associated with patient's autonomy, adherence and compliance. Patients with higher self‐efficacy may proactively take autonomous action, whereas patients with lower levels of self‐efficacy may simply follow the advice of their clinicians and in some cases may need more encouragement, reminders and feedback from their healthcare team to continue taking action. Provision of information and communication may also impact patients' autonomy and therefore their actions. While external factors impacting patient actions were not prominent in the data, it is noteworthy to mention factors such as patient's psychosocial and cultural perceptions may also influence patient's actions.

In summary, this review identifies a significant gap in the literature to understand how patient behave during the perioperative periods and what influences their actions. Specific research that assess ‘*activities*, *roles and behavioural interventions for patients and providers*’ that support patient engagement for hospital service improvement is required (Liang et al., 2017).

### 4.4 Strength and limitations

This is the first paper to systematically review qualitative and quantitative studies about how surgical patient engagement is conceptualised and operationalised during the perioperative period. To ensure the rigour and validity reporting and quality assessment, guidelines (JBI guidelines and PRISMA) in this field were followed. Study screening, data extraction, analysis and synthesis were reassessed by three reviewers. So were the quality assessment. All of the studies were of either high (16/29) or moderate (13/29). The high quality of the evidence strengthens the validity of our findings. Furthermore, the reviewers have a diverse background in qualitative, quantitative and mixed methods research and bring their knowledge and expertise from the fields of nursing, surgical, complementary medicine and implementation science. Such diverse expertise across a range of disciplines promotes the relevance and value of the findings presented. Finally, the narrowly defined perioperative time frame, allows for specific contextual data relevant to perioperative patient engagement.

This review has a few methodological limitations. First, it did not include grey literature and was restricted to publications in English. Our findings cannot be expanded to non‐English cultures. Second, the lack of standardised terms for indexing qualitative research in electronic databases meant the search relied on a purposively selected set of keywords. To address this limitation, a combination of MESH terms and appropriate text were developed and trialled before being finalised. All efforts were taken to include relevant search terms, as various terms are used interchangeably with the patient engagement term in the literature (Manafo et al., 2018). To ensure selected terms were not too constrictive, the databases were searched with the aid of a medical and a biomedical science librarian, and the search terms were pretrialled. Together with a three‐person team in data selection, extraction and analysis, this process ensures the adequacy and trustworthiness of the papers being selected. Third, this review excluded minor surgeries and those not operated under general anaesthesia. This decision was made because general anaesthesia is linked to many complications that patient engagement is essential in their management. So, our findings cannot be completely expanded to minor surgeries. Fourth, subgroup analyses were not performed due to an analytical priority on capturing common themes across the literature, as well as the diversity of studies' methodological approaches. Nevertheless, majority of the included studies did not report demographical influences in their results. Finally, as stated in the findings, a patient's *capacity to communicate* was not based on validated, psycho‐metric instruments. Rather, this subtheme was developed from subjective data. This does not necessarily discount the validity of the patients' perceptions.

While these limitations should be acknowledged, we do not believe they weaken the findings of the review.

## 5 CONCLUSION

In this research, we explored how patient engagement is conceptualised and operationalised in perioperative period. We identified a lack of explicit definitions of patient engagement in perioperative settings, which we view as problematic. The operationalisation of patient engagement in perioperative settings can broadly be classified into four distinct areas including (1) provision of information, (2) communication, (3) decision‐making and (4) action‐taking. These four elements highlight different facets of the concept of perioperative patient engagement and should be treated as interconnected and co‐dependent on each other. Future studies should address knowledge gaps in defining surgical patient engagement and examine how patient engages through following a patient's journey before and after surgery.

## 6 RELEVANCE TO CLINICAL PRACTICE

Understanding how concept of patient engagement is defined and operationalised in perioperative settings may support nurses, clinicians and healthcare administrators to implement relevant procedures that guide behaviours and create environments that promote patient engagement. Patient engagement requires behavioural changes of nurses and clinicians. An explicit conceptualisation of patient engagement will improve our shared understanding of core aspects and variations of patient engagement; and may inform pedagogies in the education of nurses and clinicians concerning actions and behaviours that may promote engagement. This review contributes to expanding the existing knowledge of patient engagement by providing a framework of surgical patient engagement during the perioperative period, through providing information, communicating with patients, implementing shared decision‐making and empowering patients to take action.

## AUTHOR CONTRIBUTIONS

ZC, ZZ, SC and WS formulated the research question and developed the protocol. ZC searched the literature, and ZC, ZZ and SC screened the studies. ZC conducted data extraction and validated by ZZ and SC. All three assessed the quality of studies, and discussed and analysed the data. ZC, ZZ, SC and WS interpreted the data. ZC drafted the manuscript. All authors have revised, commented on and reviewed the drafts and approved the final version.

## FUNDING INFORMATION

A/Prof Zhen Zheng is supported by a Translating Research into Practice fellowship from the National Health Medical Research Council (NHMRC TRIP, APP1110446). The authors of this review would also like to acknowledge the help received from librarians at Northern Hospital and RMIT University for searching research databases.

## CONFLICT OF INTEREST STATEMENT

The authors are conducting a research project to examining surgical patient engagement. This review is the stage one of the project. No other conflict of interest to be declared.

## ETHICS STATEMENT

No ethics approval is required for this systematic review.

## PATIENT CONSENT STATEMENT

No direct patient recruitment is involved in this review. This item is not applicable.

## Supporting information


Appendix S1.

Appendix S2.

Appendix S3.

## Data Availability

The data sets generated during and/or analysed during this study are available from the corresponding author upon reasonable request.
