# Supplementary material for: Patient engagement in perioperative settings: A mixed method systematic review
Source: J Clin Nurs. 2023 Apr 18;32(17-18):5865–85. doi: 10.1111/jocn.16709 (PMC10946744; doi:10.1111/jocn.16709)
Supplement: Supplementary file 1 — Appendix S1. [file JOCN-32-5865-s001.docx]

**Appendix 1 Systematic Review Search Strategies**

# Databases

- PubMed
- EMBASE
- Cochrane library
- CINAHL

# Selection criteria

## Inclusion criteria:

- Report on patient engagement or an ‘element/factor’ of patient engagement (i.e. SDM, communication, etc.)
- Focus on surgical patients
- Patients having a surgical procedure under general anaesthesia
- Clinical trials, protocols, original papers, original grey literature

## Exclusion criteria:

- Only focus on engagement in policy
- Only focus on engagement in research
- Only focus on Paediatrics
- Only focus on minor surgeries with NO General Anaesthetics
- Reviews and non-original papers

# Search terms and sample search results

**PubMed**

| # | Query | Results |
| --- | --- | --- |
| [#22](https://www-ncbi-nlm-nih-gov.ezproxy.lib.rmit.edu.au/pubmed/advanced) | Search **((((("Patient Participation"[Mesh]) OR "patient engagement") OR "consumer engagement") OR "client engagement")) AND ((((("Perioperative Period"[Mesh]) OR "Perioperative Care"[Mesh]) OR "Surgical Procedures, Operative"[Mesh])) OR "surgical patient*")** | [1840](https://www-ncbi-nlm-nih-gov.ezproxy.lib.rmit.edu.au/pubmed/?cmd=HistorySearch&querykey=22) |
| [#21](https://www-ncbi-nlm-nih-gov.ezproxy.lib.rmit.edu.au/pubmed/advanced) | Search **(((("Perioperative Period"[Mesh]) OR "Perioperative Care"[Mesh]) OR "Surgical Procedures, Operative"[Mesh])) OR "surgical patient*"** | [2897313](https://www-ncbi-nlm-nih-gov.ezproxy.lib.rmit.edu.au/pubmed/?cmd=HistorySearch&querykey=21) |
| [#20](https://www-ncbi-nlm-nih-gov.ezproxy.lib.rmit.edu.au/pubmed/advanced) | Search **"surgical patient*"** | [2759](https://www-ncbi-nlm-nih-gov.ezproxy.lib.rmit.edu.au/pubmed/?cmd=HistorySearch&querykey=20) |
| [#17](https://www-ncbi-nlm-nih-gov.ezproxy.lib.rmit.edu.au/pubmed/advanced) | Search **(("Perioperative Period"[Mesh]) OR "Perioperative Care"[Mesh]) OR "Surgical Procedures, Operative"[Mesh]** | [2896184](https://www-ncbi-nlm-nih-gov.ezproxy.lib.rmit.edu.au/pubmed/?cmd=HistorySearch&querykey=17) |
| [#16](https://www-ncbi-nlm-nih-gov.ezproxy.lib.rmit.edu.au/pubmed/advanced) | Search **"Surgical Procedures, Operative"[Mesh]** | [2892040](https://www-ncbi-nlm-nih-gov.ezproxy.lib.rmit.edu.au/pubmed/?cmd=HistorySearch&querykey=16) |
| [#13](https://www-ncbi-nlm-nih-gov.ezproxy.lib.rmit.edu.au/pubmed/advanced) | Search **"Perioperative Care"[Mesh]** | [140706](https://www-ncbi-nlm-nih-gov.ezproxy.lib.rmit.edu.au/pubmed/?cmd=HistorySearch&querykey=13) |
| [#10](https://www-ncbi-nlm-nih-gov.ezproxy.lib.rmit.edu.au/pubmed/advanced) | Search **"Perioperative Period"[Mesh]** | [77011](https://www-ncbi-nlm-nih-gov.ezproxy.lib.rmit.edu.au/pubmed/?cmd=HistorySearch&querykey=10) |
| [#6](https://www-ncbi-nlm-nih-gov.ezproxy.lib.rmit.edu.au/pubmed/advanced) | Search **((("Patient Participation"[Mesh]) OR "patient engagement") OR "consumer engagement") OR "client engagement"** | [24504](https://www-ncbi-nlm-nih-gov.ezproxy.lib.rmit.edu.au/pubmed/?cmd=HistorySearch&querykey=6) |
| [#5](https://www-ncbi-nlm-nih-gov.ezproxy.lib.rmit.edu.au/pubmed/advanced) | Search **"client engagement"** | [149](https://www-ncbi-nlm-nih-gov.ezproxy.lib.rmit.edu.au/pubmed/?cmd=HistorySearch&querykey=5) |
| [#4](https://www-ncbi-nlm-nih-gov.ezproxy.lib.rmit.edu.au/pubmed/advanced) | Search **"consumer engagement"** | [163](https://www-ncbi-nlm-nih-gov.ezproxy.lib.rmit.edu.au/pubmed/?cmd=HistorySearch&querykey=4) |
| [#3](https://www-ncbi-nlm-nih-gov.ezproxy.lib.rmit.edu.au/pubmed/advanced) | Search **"patient engagement"** | [1889](https://www-ncbi-nlm-nih-gov.ezproxy.lib.rmit.edu.au/pubmed/?cmd=HistorySearch&querykey=3) |
| [#2](https://www-ncbi-nlm-nih-gov.ezproxy.lib.rmit.edu.au/pubmed/advanced) | Search **"Patient Participation"[Mesh]** | [22838](https://www-ncbi-nlm-nih-gov.ezproxy.lib.rmit.edu.au/pubmed/?cmd=HistorySearch&querykey=2) |
