# Supplementary material for: Patient engagement in perioperative settings: A mixed method systematic review
Source: J Clin Nurs. 2023 Apr 18;32(17-18):5865–85. doi: 10.1111/jocn.16709 (PMC10946744; doi:10.1111/jocn.16709)
Supplement: Supplementary file 3 — Appendix S3. [file JOCN-32-5865-s003.docx]

**Appendix 1 Systematic Review Search Strategies**

# Databases

- PubMed
- EMBASE
- Cochrane library
- CINAHL

# Selection criteria

## Inclusion criteria:

- Report on patient engagement or an ‘element/factor’ of patient engagement (i.e. SDM, communication, etc.)
- Focus on surgical patients
- Patients having a surgical procedure under general anaesthesia
- Clinical trials, protocols, original papers, original grey literature

## Exclusion criteria:

- Only focus on engagement in policy
- Only focus on engagement in research
- Only focus on Paediatrics
- Only focus on minor surgeries with NO General Anaesthetics
- Reviews and non-original papers

# Search terms and sample search results

**PubMed**

| # | Query | Results |
| --- | --- | --- |
| [#22](https://www-ncbi-nlm-nih-gov.ezproxy.lib.rmit.edu.au/pubmed/advanced) | Search **((((("Patient Participation"[Mesh]) OR "patient engagement") OR "consumer engagement") OR "client engagement")) AND ((((("Perioperative Period"[Mesh]) OR "Perioperative Care"[Mesh]) OR "Surgical Procedures, Operative"[Mesh])) OR "surgical patient*")** | [1840](https://www-ncbi-nlm-nih-gov.ezproxy.lib.rmit.edu.au/pubmed/?cmd=HistorySearch&querykey=22) |
| [#21](https://www-ncbi-nlm-nih-gov.ezproxy.lib.rmit.edu.au/pubmed/advanced) | Search **(((("Perioperative Period"[Mesh]) OR "Perioperative Care"[Mesh]) OR "Surgical Procedures, Operative"[Mesh])) OR "surgical patient*"** | [2897313](https://www-ncbi-nlm-nih-gov.ezproxy.lib.rmit.edu.au/pubmed/?cmd=HistorySearch&querykey=21) |
| [#20](https://www-ncbi-nlm-nih-gov.ezproxy.lib.rmit.edu.au/pubmed/advanced) | Search **"surgical patient*"** | [2759](https://www-ncbi-nlm-nih-gov.ezproxy.lib.rmit.edu.au/pubmed/?cmd=HistorySearch&querykey=20) |
| [#17](https://www-ncbi-nlm-nih-gov.ezproxy.lib.rmit.edu.au/pubmed/advanced) | Search **(("Perioperative Period"[Mesh]) OR "Perioperative Care"[Mesh]) OR "Surgical Procedures, Operative"[Mesh]** | [2896184](https://www-ncbi-nlm-nih-gov.ezproxy.lib.rmit.edu.au/pubmed/?cmd=HistorySearch&querykey=17) |
| [#16](https://www-ncbi-nlm-nih-gov.ezproxy.lib.rmit.edu.au/pubmed/advanced) | Search **"Surgical Procedures, Operative"[Mesh]** | [2892040](https://www-ncbi-nlm-nih-gov.ezproxy.lib.rmit.edu.au/pubmed/?cmd=HistorySearch&querykey=16) |
| [#13](https://www-ncbi-nlm-nih-gov.ezproxy.lib.rmit.edu.au/pubmed/advanced) | Search **"Perioperative Care"[Mesh]** | [140706](https://www-ncbi-nlm-nih-gov.ezproxy.lib.rmit.edu.au/pubmed/?cmd=HistorySearch&querykey=13) |
| [#10](https://www-ncbi-nlm-nih-gov.ezproxy.lib.rmit.edu.au/pubmed/advanced) | Search **"Perioperative Period"[Mesh]** | [77011](https://www-ncbi-nlm-nih-gov.ezproxy.lib.rmit.edu.au/pubmed/?cmd=HistorySearch&querykey=10) |
| [#6](https://www-ncbi-nlm-nih-gov.ezproxy.lib.rmit.edu.au/pubmed/advanced) | Search **((("Patient Participation"[Mesh]) OR "patient engagement") OR "consumer engagement") OR "client engagement"** | [24504](https://www-ncbi-nlm-nih-gov.ezproxy.lib.rmit.edu.au/pubmed/?cmd=HistorySearch&querykey=6) |
| [#5](https://www-ncbi-nlm-nih-gov.ezproxy.lib.rmit.edu.au/pubmed/advanced) | Search **"client engagement"** | [149](https://www-ncbi-nlm-nih-gov.ezproxy.lib.rmit.edu.au/pubmed/?cmd=HistorySearch&querykey=5) |
| [#4](https://www-ncbi-nlm-nih-gov.ezproxy.lib.rmit.edu.au/pubmed/advanced) | Search **"consumer engagement"** | [163](https://www-ncbi-nlm-nih-gov.ezproxy.lib.rmit.edu.au/pubmed/?cmd=HistorySearch&querykey=4) |
| [#3](https://www-ncbi-nlm-nih-gov.ezproxy.lib.rmit.edu.au/pubmed/advanced) | Search **"patient engagement"** | [1889](https://www-ncbi-nlm-nih-gov.ezproxy.lib.rmit.edu.au/pubmed/?cmd=HistorySearch&querykey=3) |
| [#2](https://www-ncbi-nlm-nih-gov.ezproxy.lib.rmit.edu.au/pubmed/advanced) | Search **"Patient Participation"[Mesh]** | [22838](https://www-ncbi-nlm-nih-gov.ezproxy.lib.rmit.edu.au/pubmed/?cmd=HistorySearch&querykey=2) |

**Appendix 2 Four Type of Coping Behaviour (Jorgensen & Fridlund, 2016)**

|  | *Exceeding*  *boundaries of*  *capability* | *Protecting*  *boundaries of*  *capability* | *Challenging*  *boundaries of*  *capability* | *Accepting*  *boundaries*  *of capability* |
| --- | --- | --- | --- | --- |
| Description | - patient wanting to comply with expectations. - Often doing more than they were required - a sense of pride in accomplishment | - a need to be in control of their body and mind - fear of complications during and after the operation | - competitive and risk-taking personality and enjoyed comparing themselves with fellow patients. - saw early discharge as a goal and had clear plans for recovery after discharge. | - aimed to just do their best - not stressed or pressured by the expected responsibility for their own recovery |
| Relationship with HPs | health professionals had to step in and correct overexerting behaviour among these patients | Patients with this behaviour style often challenged their health professionals and commonly initiated a power struggle with their healthcare team regarding their recovery plans. | These patients were typically well prepared and informed about their role as a patient and they accepted the responsibility for their own recovery. | These patients were observed to take their time, see information that seemed sufficient to them without feeling anxious about it. |
| EQ5D-VAS | 53 | 10 | 20 | 41 |
| Anxiety | Low | High | Low | High |
| Fast-track Program | Match | Mismatch | Match | Match |
